# Fused embryos and pre-metamorphic conjoined larvae in a broadcast spawning reef coral

**DOI:** 10.12688/f1000research.6136.2

**Published:** 2015-03-05

**Authors:** Lei Jiang, Xin-Ming Lei, Sheng Liu, Hui Huang

**Affiliations:** 1 Key Laboratory of Tropical Marine Bio-resources and Ecology, South China Sea Institute of Oceanology, Chinese Academy of Sciences, Guangzhou, 510301, China; 2 Tropical Marine Biological Research Station in Hainan, Chinese Academy of Sciences, Sanya, 572000, China; 3 University of Chinese Academy of Sciences, Beijing, 100049, China

**Keywords:** fusion, conjoined larvae, spawn slicks, inborn colonies, Platygyra daedalea

## Abstract

Fusion of embryos or larvae prior to metamorphosis is rarely known to date in colonial marine organisms. Here, we document for the first time that the embryos of the broadcast spawning coral *Platygyra daedalea* could fuse during blastulation and further develop into conjoined larvae, and the settlement of conjoined larvae immediately resulted in inborn juvenile colonies. Fusion of embryos might be an adaptive strategy to form pre-metamorphic chimeric larvae and larger recruits, thereby promoting early survival. However, future studies are needed to explore whether and to what extent fusion of coral embryos occurs in the field, and fully evaluate its implications.

## Introduction

In sessile colonial marine invertebrates (e.g., sponges, cnidarians, bryozoans and ascidians), fusion among conspecifics during early ontogeny could immediately lead to a marked increase in juvenile size, thereby enhancing the performance in growth, survival and competition ^1, 2^. In addition, the allogenic fusion is expected to form chimeras which possess greater genetic variability and wider ranges of physiological resistance ^1^. Larvae of colonial marine organisms tend to settle in a gregarious manner ^3– 7^ and their juveniles often come into physical contact through growth and then fuse ^8– 10^. These life history traits increase the opportunities for fusion, and important rates of chimerism due to allogenic fusion have been detected in wild natural populations of corals and ascidians ^11, 12^. Nevertheless, fusion of embryos or larvae during planktonic and dispersive phase (i.e. prior to settlement and metamorphosis) is rarely known to date.

Modular marine invertebrates like sponges and cnidarians usually spawn their gametes in a high synchrony ^13– 15^, thus also providing the chance of contact and fusion among embryos or larvae. For instance, sticky eggs released by the oviparous sponge *Cliona celata* were found to adhere to each other and form flattened egg mass, within which larvae fused in twos or threes. The compound larvae metamorphosed into sponges with single oscula, indicating the cytomictical fusion among embryos or larvae ^13^. More recently, larvae of two sponges and sun coral *Tubastraea coccinea* have been demonstrated to fuse and generate swimming chimeras ^16– 18^. Furthermore, sexually produced embryos of a non-colonial sea anemone *Urticina feline* were observed to fuse naturally during internal brooding, generating pre-metamorphic cytomictical and sectorial chimeras ^19, 20^. These findings suggested that the direct contact between embryos and larvae would facilitate fusion either during internal brooding or pelagic phase.

For broadcast spawning corals, synchronous spawning events usually result in billions of naked embryos floating at the sea surface in the form of spawn slicks ^21, 22^. The direct contact between naked embryos highlights the possibility of fusion of coral embryos while sticking together in slicks. Moreover, previous studies have demonstrated there is a window in ontogeny, before allorecognition system matures, when newly settled polyps can fuse ^23^. Time for allorecognition maturation in reef corals varied from 4 months following settlement in brooding species ^24^, to 1–3 years in spawning species ^9, 10^. This further supports the possibility of fusion at embryonic stage when allorecognition may be weak in corals. As yet, the possible occurrence of fused embryos and conjoined larvae in broadcast spawning corals has not been investigated.

Here, we happened to test this unexplored probability of fusion of embryos in broadcast spawning reef corals. We experimentally mimicked spawn slicks using gametes collected from 4 mature colonies of *Platygyra daedalea*, and followed the fate and development of embryos within lab-generated slicks.

## Materials and methods

Ten gravid colonies of *P. daedalea* (20–30 cm in diameter) were collected at depth between 2–4 m from Luhuitou fringing reef in Sanya, China (18°12′N, 109°28′E). Corals were maintained in an outdoor tank with flowing sand-filtered seawater in Tropical Marine Biological Research Station in Hainan, Sanya. Four colonies spawned around 22:00 on May 18, 2014 (5 nights after full moon). Egg-sperm bundles were collected using pipettes, then combined and gently agitated to facilitate bundle disintegration and cross-fertilization. Fertilization was allowed to take place for about 2 hours, after which eggs (ca. 300, 000) were washed two times with fresh seawater and suspended in a 15 cm-diameter jar. Because of the logistical constraints, eggs were left undisturbed and they formed dense slicks on the seawater surface. The next morning around 08:30, embryos were inspected under a dissecting microscope and we accidentally discovered that some embryos fused. Embryos were washed and seawater was changed twice daily thereafter. Two days after fertilization, 500 larvae were randomly sampled to count the proportion of chimeric larvae. Seven days after fertilization, chips of crustose coralline algae *Hydrolithon onkodes* were used to induce the settlement of larvae and the recruits were reared in the lab at 28°C until June 26.

## Results

**Figure 1.**
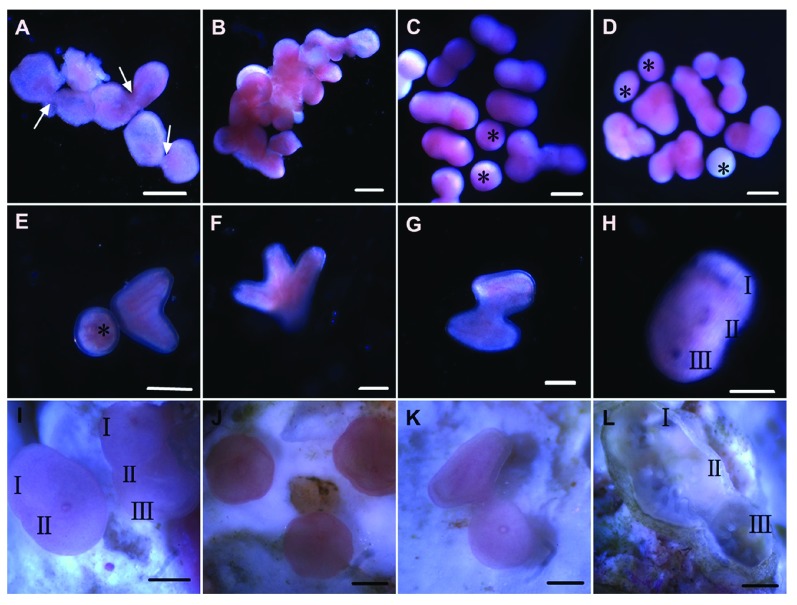
*Platygyra daedalea*. ( **A**) Fused embryos ( *arrows* point to the fusing areas). ( **B**) A dense aggregate comprising more than 20 embryos. ( **C–G**) Unitary ( *asterisks*) and conjoined larvae. ( **H, I**) Inborn colonies. ( **J**) Single settlers. ( **K**) Incomplete settlement of perpendicularly bi-fused larvae, with the left partner being parallel to the substrate. ( **L**) An inborn colony 26 days post-settlement. Roman numbers indicate visible individuals within inborn colonies. *Scale bars* 250 μm.

Embryos became bowl shaped (cushion stage) 8 h after fertilization. Notably, some embryos fused ( Figure 1A) and a substantial proportion even stuck together into dense aggregates ( Figure 1B). It could be deduced that fusion of embryos took place some time during blastulation. Mortality of embryos within the first 2 days was extremely high (>50%) and the dense aggregates all died and decomposed. Unitary larvae became pear-shaped and began to rotate actively 20 h after fertilization, while conjoined larvae were highly variable in shape. Bi-fused larvae were dominantly peanut-shaped, and multi-fused larvae were arranged in chains or triangles, or in the form of the letter “T” or “L” ( Figure 1C, D).

Of the 500 randomly sampled larvae, 174 (34.8%) were conjoined with 2–4 partners. Conjoined larvae clearly showed their spatial arrangement after elongation and fusion was apparently without polarity. Larvae either joined at the aboral end ( Figure 1E, F), or united side by side ( Figure 1G), or even fused perpendicularly ( Figure 1K). Furthermore, 56 out of the 174 conjoined larvae (32.2%) united at the aboral extremity and only these larvae were potentially competent to metamorphose normally into inborn colonies ( Figure 1H, I), which were prominently larger in size than the single settlers ( Figure 1J). In contrast, perpendicularly bi-fused larvae settled incompletely, with one partner metamorphosing and firmly attaching while the other still being parallel to the substrate and not able to settle ( Figure 1K), ultimately leading to the death of the whole entity 3 days later. Since the coralline algae provided here was not suitable for the settlement of *P. daedalea* larvae, only 12 inborn colonies were obtained in total and they persisted for 26 days post-settlement when the study ended ( Figure 1L).

## Discussion

The present study documented for the first time the fusion of embryos and inborn colonies in a broadcast spawning coral. Fusion of *P. daedalea* embryos was spontaneous, resulting simply from the aggregation and contact of embryos in mimicked slicks, which was analogous with that in sponge *C. celata* ^13^. While unlike the cytomictical compound larvae in sponge *C. celata*, the chimeric *P. daedalea* larvae were multi-headed, suggesting sectorial fusion of coral embryos and supporting the assumption that corals typically exhibit sectorial fusion ^1^.

Corals often spawn during seasonally calm periods and low-amplitude tides ^21, 22, 25^ and spawn slicks extending up to few km in length were often observed in the field ^21, 26^. Given that slicks remained aggregated 1–2 d after spawning ^21, 22^ and embryos can fuse during embryogenesis within 8 h post-fertilization, fusion of coral embryos is highly favored *in situ*. On the other hand, although mass coral spawning events usually involved several species, significant temporal differences in spawning to ensure fertilization and reproductive isolation have been demonstrated for many sympatric species ^27– 29^, which considerably increase the encounters between embryos of the same species in slicks. Taken together, fusion of coral embryos might be a naturally occurring phenomenon. However, the density of embryos here was 1700 cm ^-2^ and likely to be much higher than that in the field. Moreover, water turbulence that the embryos would experience was absent in this study. Therefore, it is possible that our experimental conditions eventually led to the formation of embryonic chimeras. Likewise, larvae brooded by *T. coccinea*, when kept at high density in still water, could metamorphose and aggregate in clusters with extended lifespan ^18^. Thus, whether fusion of coral embryos occurs in natural spawn slicks and the dispersal potential of these chimeric larvae remain to be determined.

At last, an important observation was that the chimeric larvae were able to settle firmly and form inborn colonies. The inborn colonies here originated from fusion of embryos and settlement of chimeric larvae, contrasting the traditional concept that the asexual budding of the primary polyp leads to the formation of a young coral colony ^30^, and thus fusion of embryos could be an unexpected shortcut to colony formation in reef corals. Furthermore, the inborn colonies persisted for about one month and exhibited no sign of rejection, suggesting the possibility that the embryonic chimeras might contribute to recruitment in the natural environment ^17^. These facts raise questions as to the ecological implications of inborn colonies formed as a consequence of fusion of embryos in corals. Firstly, larger coral colonies composed of multiple fused partners are known to yield remarkable gained benefits, such as enhanced survival and growth ^5, 8^. Hence, the larger initial size and the status of multi-polyp at settlement may confer these inborn colonies better capacities to compete for space and survive partial mortality.

Fusion of coral embryos also shed new light on the chimerism in scleractinian corals, which was often attributed to fusion of gregariously settling larvae ^5, 7^, or of juveniles that come into contact through growth ^7, 9, 10^. However, our study documented fusion between individuals in *P. daedalea* occurred at the embryonic stage, earlier than any other corals studied to date. Since the embryos here were produced sexually from 4 parent colonies and they were genetically distinct, fusion of embryos could be a novel mechanism for chimerism in scleractinian corals. In that case, the increased genetic diversity within these inborn colonies may translate into versatile physiological qualities, thus enabling them to better cope with environmental changes unless negative interactions occur ^1, 31, 32^.

Overall, this is the first report of embryonic chimeras in reef corals. Fusion of coral embryos could be an adaptive strategy to form larger and chimeric recruits, thereby promoting growth and survival during the vulnerable early stages ^5, 8^. Clearly, future studies are required to explore whether fusion of embryos occurs in the field and fully evaluate its biological and ecological implications.

## Ethics statement

Coral sampling was permitted by the Administration of Sanya Coral Reef National Nature Reserve, the Department of Ocean and Fisheries of Hainan Province.
