# Supplementary material for: Fused embryos and pre-metamorphic conjoined larvae in a broadcast spawning reef coral
Source: F1000Res. 2015 Mar 5;4:44. Originally published 2015 Feb 13. [Version 2] doi: 10.12688/f1000research.6136.2 (PMC4392822; doi:10.12688/f1000research.6136.2)
Supplement: Peer review history

# Peer review: Referee response for version 2

**DOI:** 10.5256/f1000research.6649.r8208

**Published:** 2015-04-02

**Authors:** Baruch Rinkevich

**Affiliations:** 1 Israel Oceanography & Limnological Research, National Institute of Oceanography, Haifa, Israel

I have read this submission. I believe that I have an appropriate level of expertise to confirm that it is of an acceptable scientific standard.

# Peer review: Referee response for version 1

**DOI:** 10.5256/f1000research.6575.r7679

**Published:** 2015-02-17

**Authors:** Baruch Rinkevich

**Affiliations:** 1 Israel Oceanography & Limnological Research, National Institute of Oceanography, Haifa, Israel

This short research article by Jiang *et al.* is timely, adds to our understanding on the magnitude of natural chimerism, and meets research standards with **methodological details that made available **to allow others to replicate this study. I recommend indexation pending responding on the below minor editorial/clarification points:

- Abstract (line 2)- An unclear sentence. Suggest revising from: ‘Here, we observed for the first time the embryos….’ to ‘Here, we document for the first time that embryos…’. See also below the note appended to the citation of Mizrahi et al., 2014 and define your statement, ’for the first time’.
- ‘Conjoined larvae’ (as from Abstract line 4 and along the manuscript). This term and the following terms ‘chimera’ and ‘fusion’ are mutually used, irrespective to the somehow different biological statuses they present (for example, ‘conjoined’ is a very sterile term indicating being, coming, or brought together so as to meet, touch, overlap, or unit). I would suggest to use the term ‘Conjoined larvae’ in the Results section when first describing the process of larval joining and then use the terms ‘fusion’ and ‘chimerism’ in all other parts of the manuscript to state the biological outcome.
- Related to the aforementioned- it will be most valuable to show evidence for the ‘fusion’ outcomes, such as histological sections, or that no disjointing between the conjoined larvae occurred following the employment of physical force.

- Introduction and Discussion- There are four papers that I would suggest the authors to add and to cite. The first and the most important is the study by Mizrahi *et al.* (2014). In this paper the authors showed that *Tubastraea coccinea* offspring can metamorphose and aggregate/fuse in groups of up to eight polyps in the water column, before settlement. This may also change the way Jiang *et al.* refer to their finding (statements such as ‘the first time’ should be revised). The second paper is the study on sponge chimeras by Gauthier and Degnan (2008). This paper reveals the potential intermixing of cells between fused partners. The third paper is the old report by Duerden, the first observation documenting ‘aggregated colonies’ ( Duerden,1902). It will also be valuable to cite Rinkevich (2011).
- Introduction 2 ^nd^ paragraph, 1 ^st^ sentence: ‘Modular marine invertebrates …. tend to aggregate after release’ is redundant to the former introduction text. Delete or rephrase.
- M & M:  ‘… then mixed and gently agitated…’ Specify if the mixed eggs/sperm were from the same genotype or from all/several genotypes.
- M & M:  ‘…to count the proportion of each type of fusion’. Which ‘types’ of fusion the authors imply to? Detail/explain.
- Discussion, below Fig 1: ‘… *P. daedalea *larvae still retained a degree of individuality…’. What is the meaning of ‘individuality’ here? What is the meaning of ‘a degree’? Morphologically? Physiologically? Define and rephrase.
- Discussion, the sentence: ‘It should be pointed out that though embryos here were produced sexually from 4 parent colonies, it did not denote they were genetically distinct’. Something is wrong with the sentence. If larvae are sexually produced they should be genetically distinct from each other. Rewrite.

I have read this submission. I believe that I have an appropriate level of expertise to confirm that it is of an acceptable scientific standard, however I have significant reservations, as outlined above.

# Response

**Published:** 2015-02-26

**Authors:** Lei Jiang

**Affiliations:** Chinese Academy of Sciences, China

- We revised the sentence in abstract as suggested. Our observation presented the embryonic chimeras as a result of fusion of embryos in a broadcast spawning coral, whereas the results of Mizrahi *et al.*, (2014) revealed that the larvae of brooding coral *Tubastraea coccinea *could metamorphose and aggregate in swimming groups. Thus we did document for the first time the fusion between individuals at the embryonic stage in reef corals. Moreover, we followed the settlement and growth of these chimeric larvae. Overall, it is proper and discreet to state “for the first time”. 
- We have followed the reviewer’s advice to use “conjoined larvae” in the Results and Abstract sections where described the larval joining. 
- This was only an accidental observation and it was a pity that we did not preserve samples for histological sections, nor did we employ physical force to see whether the conjoined larvae would disjoint. 
- As aforementioned, we did observe the embryonic chimeras and follow the settlement and post-settlement growth of chimeric larvae in a broadcast spawning coral for the first time. Mizrahi *et al.* (2014) revealed that the swimming polyp clusters survived longer and did not confirm whether these clusters can settle. Therefore, the findings of Mizrahi *et al.* (2014) were not contradictory to our statement of “the first time”. We have added the suggested references. But as for Rinkevich (2011), we think it is more suitable to cite the classical reference of Rinkevich *et al. *(1987) which mainly focused on the chimerism in colonial marine invertebrates.
- We deleted the latter part of this sentence and made it more specific as to the synchrony in gametes release.
- We used the word “combined” to show that the bundles from 4 colonies were brought together for cross fertilization.
- We replaced “each type of fusion” with “chimeric larvae”.
- We made it more specifically that these conjoined larvae were multi-headed to demonstrate the sectorial fusion.
- We were sorry for this mistake because we were a little confused at first when referring to the results of Puill-Stephan *et al*. (2012). They found that fusion between sexually produced larvae resulted in non-chemiric colonies of one genotype. Thus we got a misunderstanding that the sexually produced larvae were not genetically distinct absolutely. However, they only use nine microsatellite loci to genotype. We have corrected and rewritten this part.

# Peer review: Referee response for version 1

**DOI:** 10.5256/f1000research.6575.r7666

**Published:** 2015-02-16

**Authors:** Andrew Baird

**Affiliations:** 1 School of Marine Biology and Aquaculture, James Cook University, Townsville, QLD, Australia

This is well written article and an interesting observation although some of the literature cited is a little dated (e.g. Harrison & Wallace 1990). To my knowledge this is first time that anyone has followed these embryonic chimeras through to settlement and that fact that they can settle and grow is an important observation. However, embryonic chimera formation is an artifact of the experimental conditions i.e. the very high densities of embryos in still water in the bowls. Indeed, embryonic chimera formation is quite common, particularly when working with larvae of merulinds at higher temperatures. It is very rare to see chimeras in spawn slicks or on settlement tiles, at least on soaks of short duration, although this has yet to be quantified. Therefore, embryonic chimera formation is highly unlikely to be of much ecological or evolutionary significance. Indeed, a phenonomen of much more ecological significance is the fact that many embryos break up during development under conditions likely to prevail in the wild (see Heyward and Negri, 2012).

I have read this submission. I believe that I have an appropriate level of expertise to confirm that it is of an acceptable scientific standard.

# Response

**Published:** 2015-02-26

**Authors:** Lei Jiang

**Affiliations:** Chinese Academy of Sciences, China

Spawn slicks are commonly observed in the field and more importantly, they remained aggregated 1-2 days after spawning (Oliver *et al*., 1987; Gilmour *et al*., 2009). It is true that these conditions are favorable for the formation of embryonic chimeras. Moreover, the high density and absence of water turbulence in this study might trigger the fusion of embryos. Under similar experimental conditions, larvae of *A. millepora* tended to settle in aggregation (Puill-Stephan *et al.*, 2012), and larvae of *T. coccinea* formed swimming polyp clusters ( Mizrahi et al. (2014)). Therefore, though fusion of coral embryos has never been reported in the wild, we speculate it might occur in the field and remains to be determined especially corals often broadcast spawn their gametes during calm periods and low-amplitude tides.
